# Digital insights: bridging the phenotype-to-genotype divide

**DOI:** 10.1093/jxb/erab108

**Published:** 2021-04-02

**Authors:** Matthew F McCabe, Mark Tester

**Affiliations:** 1 Hydrology, Agriculture and Land Observation (HALO) Laboratory, Water Desalination and Reuse Center, Division of Biological and Environmental Sciences and Engineering, King Abdullah University of Science and Technology, Thuwal, Saudi Arabia; 2 Center for Desert Agriculture, Division of Biological and Environmental Sciences and Engineering, King Abdullah University of Science and Technology, Thuwal, Saudi Arabia

**Keywords:** Digital agriculture, drones, genome-wide association studies, machine learning, phenotype, genotype, remote sensing, unmanned aerial vehicles

## Abstract

This article comments on:

**Han R, Wong AJY, Tang Z, Truco MJ, Lavelle DO, Kozik A, Jin Y, Michelmore R**. 2021. Drone phenotyping and machine learning enable discovery of loci regulating daily floral opening in lettuce. Journal of Experimental Botany **72,**2979–2994.

**The convergence of autonomous platforms for field-based phenotyping with advances in machine learning for big data analytics and rapid sequencing for genome description herald the promise of new insights and discoveries in the plant sciences. Han *et al.* (2021) leverage these emerging tools to navigate the challenging path from field-based mapping of phenotypic features to identifying specific genetic loci in the laboratory: in this case, loci responsible for regulating daily flowering time in lettuce. While their contribution neatly illustrates these exciting technological developments, it also highlights the work that remains to bridge these multidisciplinary fields to more fully deliver upon the promise of digital agriculture.**

With growing populations driving an increased demand for food over the next few decades (Foley *et al.*, 2011), combined with changes in climate that present as threat multipliers (Wheeler and von Braun, 2013), there is an immediate need to develop tools and techniques for enhancing the performance of our agricultural systems (Tester and Langridge, 2010). Digital agriculture is at the forefront of this effort (Shepherd *et al.*, 2020). In broad terms, this encompasses the collection, analysis, and interpretation of data across the food production system, using a variety of approaches to turn gathered information into actionable knowledge. At its core, digital agriculture is simply a data mining activity, where the output is enhanced knowledge on crop condition or physiological behaviour, as well as insights into on-farm responses to environmental changes. The expectation is that a data-driven approach to agricultural production has the potential to deliver a more sustainable utilization and management of resources, while increasing the output of farms across diverse locations and environments (Basso and Antle, 2020).

A key element of digital agriculture is the procurement of high-resolution spatial and temporal data in a timely and useable manner. Indeed, the promise of remote sensing for improving the characterization and description of this field has been evident for many decades (McCabe *et al.*, 2017). Unfortunately, it is one that has remained largely unfulfilled, principally as a consequence of inadequate spatial detail. However, the recent rise of unmanned aerial vehicles (UAVs) has witnessed a revolution in the way spatial information can be obtained and interpreted, offering capacity for on-demand sensing with high spatio-temporal coverage (Maes and Steppe, 2019). For UAVs, a wide range of sensing payloads have become available (see Box 1), ranging from traditional optical red–green–blue (RGB) systems, to advanced hyperspectral and LIDAR sensors, offering the possibility of derivable metrics that can be processed in near real-time and at spatially explicit scales. With such flexibility, unique fusions of optical, thermal, and multi- and hyperspectral sensors facilitate the retrieval of numerous land surface parameters and variables, spanning canopy structural properties, vegetation health and condition, soil and leaf temperatures, and even higher order variables such as evaporation and transpiration (Manfreda *et al.*, 2018).

Box 1 **. Unmanned aerial vehicles for agricultural remote sensing**

Given its capacity for local-to-global coverage, remote sensing has an obvious role in advancing precision agriculture and plant phenotyping studies (Weiss *et al.*, 2020). However, satellite-based observations are constrained by issues of temporal frequency and spatial resolution, as well as the latency with which data can be obtained. Although commercial high-resolution satellite data are increasingly available for precision agricultural applications (Houborg and McCabe, 2018), it is not yet at the resolution to deliver the type of plant-to-leaf scale retrievals needed for phenotyping studies (McCabe *et al.*, 2017). On the other hand, the increasing availability of flight-stable and user-friendly unmanned aerial vehicle (UAV) platforms that can be combined with lightweight miniaturized sensors has dramatically expanded the capacity for these types of focused investigations (Mahlein, 2016; Yang *et al.*, 2017). UAVs offer the possibility of ultra-high (subcentimetre) resolution sensing with on-demand sampling, realizing a considerable advantage over satellite-based sensing. An expanding array of sensing systems include combinations of thermal (Khanal et al., 2017), multispectral, and hyperspectral sensors (Aasen *et al.*, 2018), which facilitate the retrieval of a variety of vegetation indices, pigment-based retrievals, and plant structural properties (Berni *et al.*, 2009). Other sensing systems include active LIDAR for detailed structural mapping (Madec *et al.*, 2017) and even fluorescence systems that provide a means for monitoring photosynthesis and stress (Zarco-Tejada *et al.*, 2012). In combination with field-based sampling and laboratory-based analysis, UAV and related phenotyping technologies are poised to deliver knowledge advances not just to scientists but, more importantly, to farmers and breeders (Hickey *et al.*, 2019).

As a consequence of these developments, we are increasingly awash with rich geospatial datasets. Yet data availability is only one aspect of the knowledge equation. To realize impact, we must also develop the tools and techniques that can turn this information into usable and useful products. New and improved retrieval algorithms, advanced data-fusion and data-harmonization approaches, and, increasingly, machine learning techniques, are all required. However, so too are more fundamental assessments that relate these varied observations to detailed *in situ* collections, providing a basis to drive improvements in process descriptions and allowing new mechanistic relationships to be developed, as well as improved understanding of underlying biological responses to environmental change. The opportunity to exploit remotely sensed features in the field, and then use these to identify key genetic determinants of phenotypes in the laboratory, represents one of the latest and (potentially) most impactful of frontiers (Shi *et al.*, 2016).

In the case of Han *et al.* (2021), remote sensing-driven insights support a detailed genetics-based investigation of a key phenotypic characteristic, namely differential floral opening and closing times. Using a multitemporal collection of UAV-derived RGB imagery (and supported by some prior ground-based photo and video sequences), the authors were able to produce a training–testing dataset to be used as input into several machine learning approaches, with the ultimate aim of automatically classifying the spatio-temporal behaviour of numerous individual lettuce plants within a field, and then to link the observed response to subsequent genetic analyses. Although RGB imagery was used in this example, there is an extensive and growing body of work devoted to the retrieval of specific plant spectral characteristics that may offer additional insights into patterns of plant growth and response. How these multiple plant functional traits and inter-related descriptors can be used to provide even deeper insights into phenotyping (and genotyping) studies represents an exciting area of research.

Increasingly, we are seeing the implementation of machine learning approaches driving these insights (Bauer *et al.*, 2019). Machine learning has already proven to be a versatile tool to assist in unlocking the vast volumes of both remote sensing (Zhu *et al.*, 2017) and genomic data (Eraslan *et al.*, 2019). However, its application routinely presents its own challenges. An aspect of this was investigated by the authors via their considered assessment of several learning approaches. The rationale for doing this is sound since, at this stage, it remains unclear under what circumstances and pre-conditions a specific machine learning technique is best suited for use in any particular application; that is, it is unusual to know the optimal approach to apply *a priori*. Reproducibility represents another aspect requiring careful attention: the need for thorough documentation of parameter selection (and the underlying justification for these), let alone the myriad other tuning elements available to the user, highlights the need for thorough description. Finally, the training and accuracy assessments required in machine learning are critical to understanding their efficacy. Confusion matrices, classification errors, sensitivity, specificity, and false-positive rates all influence the relative worth of any particular approach. A high prediction accuracy can be achieved by many different means, and for many different reasons: it may indicate either a robust, or an overly tuned, highly specific model. Resolving the conflict between model generality and transferability remains a key challenge not limited to the plant sciences (Yosinski *et al.*, 2014).

Just as phenotyping of field-grown plants is being significantly enhanced by remote sensing and machine learning, so genotyping is being significantly enhanced by the power of high-throughput sequencing and the rapid proliferation of high-quality genome sequences. Phenotyping in controlled environments still has an important role, especially to investigate the effects on plants of manipulations to the environment; however, the value of field phenotyping is, of course, that measured traits are more likely to be more directly relevant to field-grown crops, and thus of use to breeders and agronomists. Similarly, on the genotyping side, as gold and platinum standard reference genomes of crops become increasingly available, and whole-genome re-sequencing becomes trivially cheap, so the need to use model species reduces. We are living in exciting times for plant science.

UAV imaging and analyses are ideal for field trials of mapping populations, where ultra-high spatial and temporal resolution information that cannot be manually collected in the field is so valuable. However, one aspect that requires increased attention is the use of sensors and Internet-of-Things (IoT) approaches to accurately describe the environment in which the plants are growing (Papoutsoglou *et al.*, 2020). In a similar vein, we need to remain vigilant to the often strong effects of genotype–environment (G×E) interactions, and the need to repeat experiments across more than one season. An extra layer on top of the work of Han *et al*. would be to identify where genetic controls of traits were stable across seasons, and where loci had different effects in the face of different temperatures and day-lengths, for example. This could be particularly important for lettuce, where flowering time is well known to be strongly affected by these environmental parameters.

It should also be noted that genetic studies could be considered to be ‘simply’ the (admittedly sophisticated) positing of hypotheses, and that such work needs to be built upon with the testing of the effects of candidate genes on plant phenotype. In a diversity panel, for example, this could start with a haplotype analysis of candidate genes, where differences in phenotypes are correlated with different alleles of candidate genes. In a bi-parental population, such as used by Han *et al*., fine mapping approaches can be used. Ultimately, testing of candidate gene function is usually done using reverse genetic approaches, such as gene editing to knock out gene function.

## The path forward

While discoveries and insights are often (waiting) to be found within the overlapping boundaries of aligned disciplines, it is a domain that presents more than just knowledge-related barriers. Language and terminology present as real constraints, and the need for researchers to be conversant in multiple fields is paramount. This is certainly the case with applications of machine learning to various disciplines (e.g. remote sensing or plant sciences), and even more so when multiple disciplines converge, as we see in Han *et al.* (2021). The availability of these emerging technologies and techniques, and their cross-field potential, is encouraging this dialogue, which can only be of benefit to all fields of investigation. Still, it remains to be seen whether the information revolution that is occurring within the agricultural sector will resolve to the scales necessary to realize change at the level of the farm or the seed company, or whether the researcher to end-user divide will remain. Perhaps more importantly, it is imperative that these technologies do not just produce more data and information, but lead to actual insight and knowledge. Developing use cases and objectively demonstrating the information benefit, as is done in Han *et al.* (2021), represents an important step towards realizing this goal.
